# Sputum biomarkers in IPF: Evidence for raised gene expression and protein level of IGFBP-2, IL-8 and MMP-7

**DOI:** 10.1371/journal.pone.0171344

**Published:** 2017-02-08

**Authors:** J. Guiot, M. Henket, J. L. Corhay, C. Moermans, R. Louis

**Affiliations:** Pneumology Department, CHU Liège, Liège, Belgium, CHU Liège, Pneumology Department, Domaine universitaire du Sart-Tilman, Liège, Belgium; University of Giessen Lung Center, GERMANY

## Abstract

**Background:**

Idiopathic pulmonary fibrosis (IPF) is a rare lung disease of unknown origin leading rapidly to death. This paper addresses the issue of whether sputum induction is a suitable tool to study respiratory tract inflammation and potential biomarkers in IPF compared to COPD, a fibrosing airway wall disease.

**Methods:**

In a cross-sectional analysis, 15 IPF patients, 32 COPD and 30 healthy subjects underwent sputum induction. Total sputum cell counts and the amount of TGF- β, IGF-1, IGF-2, IGFBP-1, IGFBP-2, IGFBP-3, IL-8, IL-13, MMP-7, MMP-9, YKL-40, TNF-α and KL-6 in sputum supernatant were analysed. We also profiled gene expression of cells in the induced sputum for TGF-β, MMP-7, YKL-40, IGFBP-2, IL-6, IL-8 and TNF-α.

**Results:**

IPF patients, like COPD, had increased sputum absolute number of neutrophils, eosinophils, macrophages and epithelial cells compared to HS. IPF sputum supernatants had increased concentrations of IGFBP-2, IL-8, TGF-β, MMP-7, MMP-9 and KL-6 (p<0.05, p<0.0001, p<0.05, p<0.05, p<0.0001, p<0.05 respectively) when compared to healthy subjects where COPD had higher IL-6 and TNF-α levels than IPF (p<0.05 and p<0.05 respectively) and HS (p<0.0001 and p<0.001 respectively) and higher IL-8 and MMP-9 than HS (p<0.0001 and p<0.001 respectively). Conversely to IL-6 and TNF-α, MMP-7 was increased in IPF compared to COPD (p<0.05). The KL-6 and MMP-7 protein levels in sputum were inversely correlated with total lung capacity (TLC, % of predicted) in IPF patients (r = -0.73 and r = -0.53 respectively). Sputum gene expression analysis identified a significant increase for IGFBP-2, IL-6, IL-8 and MMP-7 in IPF compared to HS (p<0.05, p<0.01, p<0.05 and p<0.0001 respectively) and for IGFBP-2, YKL-40, IL-6, IL-8 and MMP-7 compared to COPD (p<0.01, p<0.01, p<0.05, p<0.01 and p<0.0001 respectively). Furthermore, gene expression of TGF-β was increased in IPF compared to COPD (p<0.001) but not to HS.

**Conclusion:**

Our data show clear increase in expression and production of IGFBP-2, IL-8 and MMP-7 in sputum from patients with IPF that may contribute to the disease.

## Introduction

Idiopathic pulmonary fibrosis (IPF) is a rare lung disease of unknown origin leading rapidly to death [1]. The diagnostic approach is complex and requires a multidisciplinary discussion [1]. Although bronchoalveolar lavage (BAL) is an important tool for evaluating interstitial lung diseases (ILDs), induced sputum, which has demonstrated clinical interest in airway diseases [2], has been proposed as a less invasive alternative [2–4]. The research with sputum in ILDs has mainly focused on the cellularity so far and there has been few studies that have investigated the molecular inflammatory pathways in IPF using sputum analysis [5].

Many biomarkers have already been studied both in serum and BAL from ILDs and in particular in IPF where inflammation seems to take part of the disease [6] in addition to the remodelling process [7]. Among those we can identify surfactant protein A or SP-A, surfactant protein D or SP-D which has recently be identified to be correlated with pulmonary function and mortality in IPF [8], the Krebs von den Lungen 6 or KL-6, type A immunoglobulin or IgA, periostin [9–13], insulin-like growth factor binding protein or IGFBP-2 [14] and a chitinase-3-like-1 human cartilage glycoprotein or YKL-40 [15–16] which were found to be increased in serum from IPF patients. As for BAL, matrix metalloproteinase-7 (MMP-7) [17] and YKL-40 [15] were the main markers found to be raised in IPF compared to healthy subjects.

The aim of our study was to evaluate the potential of measuring biomarkers of the fibrosing process in sputum from patients with IPF. As comparators we have recruited a group of COPD, recognized to be an airway disease featuring fibrosis of the airway wall at the periphery of the lung [18].

## Materials and methods

We analysed the induced sputum of patients with IPF (n = 15) in comparison to healthy subjects (n = 30) and COPD (n = 32). The diagnosis of IPF was made according to the international recommendations of the ATS [1] using the respiratory function, HRCT scan, BAL (when available), as well as the clinical history of the patient. We excluded all other causes of ILD (such as asbestosis, hypersensitivity pneumonitis, pneumopathy associated with connective tissue diseases or toxic pneumonitis). All cases were discussed in our multidisciplinary discussion team about interstitial lung diseases composed of: a pulmonologist, a specialist in pulmonary rehabilitation, a rheumatologist, a radiologist, a pathologist, a specialist in occupational medicine. COPD were diagnosed according to the GOLD [19] and healthy subjects were recruited by advertisement in ambulatory care waiting room of the hospital. They all denied any chronic respiratory disease and had FEV1 and FVC values above 80% predicted.

The protocol was approved by the ethics committee of CHU Liège, and all subjects gave written consent before their enrolment (Belgian number: B707201422832; ref: 2014/302).

### Sputum induction and processing

After premedication with 400 micrograms inhaled salbutamol, sputum was induced by inhalation of hypertonic (NaCl 5%) or isotonic (NaCl 0.9%) saline according to the FEV1 value (> or < than 65% predicted). Saline was combined with additional salbutamol delivered by an ultrasonic nebulizer (Ultra-Neb 2000; Devilbiss, Somerset, PA, USA) with an output set at 0.9 ml/min. Each subject inhaled the aerosol for three consecutive periods of 5 min for a total of 15 min. For safety reasons, FEV1 was monitored throughout the induction and it was stopped if FEV1 fell by more than 20% from baseline. The whole sputum was collected in a plastic container, weighed, and homogenized by adding three volumes of phosphate-buffered saline (PBS), vortexed for 30 s, and centrifuged at 800g for 10 min at 4°C. Supernatant was separated from cell pellet and stored at − 80°C. The cells were resuspended in a solution containing 5mM dithiothreitol without Ca^++^ and Mg^++^ and gently rocked for 20 min at room temperature. The cell suspension was then centrifuged again at 400g at 4°C for 10 min. Squamous cells, total cell counts and cell viability checked by trypan blue exclusion were performed with a manual hemocytometer [20]. The differential leukocyte count was obtained using a cytospin stained with Rapi Diff II (Atom Scientific, Manchester UK) on 500 non squamous cells.

### Biomarkers measurements in the induced sputum

We analysed several biomarkers assumed to be critical growth factors or chemokines in the induced sputum. The concentration of IL-6, IL-13, TNF-α, MMP-7, YKL-40 were assessed by ELISA multiplex using Fluorokine® Multianalyte Profiling Kits (R and D Systems, Minneapolis, MN, USA) according to the manufacturer’s instructions. The detection limit for this assays were 7-25-7-39-61 pg/ml respectively The concentration of the other proteins were measured separately by ELISA: TGF- β, MMP-9, IL-8, IGF-1, IGFBP-1, IGFBP-2 and IGFBP-3 (DuoSet kit, R and D systems); IGF-2 (Mediagnost, Reutlingen, Germany); KL-6 (Lumipulse G KL-6 Fujirebio Europe). The detection limits for these kits were 7-25-25-32-30-125-130-450pg/ml and 100 U/ml respectively.

### Gene expression in the induced sputum

PCR was used to profile gene expression of cells in the induced sputum for TGF–β, MMP-7, YKL-40, IGFBP-2, IL-6, IL-8 and TNF-α in a subgroup of patients (HS n = 14; COPD n = 15, IPF n = 12). mRNA expression was measured in total sputum cells by Taqman reverse transcription quantitative polymerase chain reaction (RT-qPCR). RNA extraction, and RT-qPCR were performed according to description of da Silva et al [21]. The house keeping gene rRNA 18S was used as the reference gene and the fold change was calculated from the median of healthy subject group. Sequences of primers and probes used are listed in Table 1. All Probes were labeled with reporter and double-quencher dyes 5′6-carboxyfluorescein/ZEN/3′ Iowa Black FQ (5′6-FAM/ZEN/3′IBFQ). Probes and primers were synthesized by IDT (Integrated DNA Technologies, Inc., Coralville, IA).

**Table 1:** Sequence of primers and probes.

Gene name	Accession number		sequence
**IGFBP-2**	NM 000597.2	Forward	TTC ACA CAC CAG CAC TCC
		reverse	ACC TCT ACT CCC TGC ACA T
		Probe	AGC ATG GCC TGT ACA ACC TCA AAC A
**TGF-β**	NM 000660.6	Forward	GTT CAG GTA CCG CTT CTC G
		reverse	CCG ACT ACT ACG CCA AGG A
		Probe	ACC CGC GTG CTA ATG GTG GAA
**MMP-7**	NM 002423.4	Forward	GAA TGT CCC ATA CCC AAA GAA TG
		reverse	GAT GAG GAT GAA CGC TGG A
		Probe	CAT ACA GGA AGT TAA TCC CTA GAC TGC TAC CA
**IL-6**	NM 000600.4	Forward	CCA GGA GCC CAG CTA TGA AC
		reverse	CCC AGG GAG AAG GCA ACT G
		Probe	CCT TCT CCA CAA GCG CCT TCG GT
**IL-8**	NM 000584.3	Forward	CTG GCC GTG GCT CTC TTG
		reverse	CCT TGG CAA AAC TGC ACC TT
		Probe	CAG CCT TCC TGA TTT CTG CAG CTC TGT GT
**YKL-40**	NM 001276.2	Forward	TCT GGG TGT TGG AGG CTA T
		reverse	GCT CAA CAC ACT CAA GAA CAG
		Probe	TGT CTG TCG GAG GAT GGA ACT TTG G
**TNF-α**	NM 000594.3	Forward	TCA GCT TGA GGG TTT GCT AC
		reverse	TGC ACT TTG GAG TGA TCG G
		Probe	AGA TGA TCT GAC TGC CTG GGC C
**18s (rRNA)**	x03205.1	Forward	CGC CGC TAG AGG TGA AAT TCT
		reverse	CAT TCT TGG CAA ATG CTT TCG
		Probe	ACC GGC GCA AGA CGG ACC AGA

Primers and probes for 18S rRNA, IL-6 and IL-8 were designed by Gielen and al (22).

PrimeTime^®^ qPCR Assays provided by IDT (Integrated DNA Technologies, Inc., Coralville, IA) were used for IGFBP-2, MMP-7, TNF-α, TGF-β and YKL-40.

Primers and probes for 18S rRNA, IL-6 and IL-8 were designed by Gielen and al. [22]. The sequences are listed in Table 1. PrimeTime^®^ qPCR Assays provide by IDT (Integrated DNA Technologies, Inc., Coralville, IA) were used for IGFBP-2, MMP-7, TNF-α, TGF-β1 and YKL-40. We only analysed samples with more than 50% of viability with less than 30% squamous cells. We chose a cut-off of 20 CT for the 18S and excluded samples in which more than 20 CT was needed to pick up 18S.

### Pulmonary function tests

Lung function tests were performed in our routine respiratory laboratory at CHU Liège. All spirometric tests performed for this study were measured using the pneumotachograph JaegerMasterlab system (Erich Jaeger GmbH, Wuzburg, Germany). The expiratory volume in one second (FEV) and forced vital capacity (FVC) were measured in accordance with the recommendations of the European Respiratory Society (ERS)[23]. The results are expressed in ml and as percentage of predicted normal values. The Tiffeneau index or FEV / FVC is expressed as percentage. The diffusion capacity of CO (DLCO) and the ratio DLCO / VA were measured by the single breath testing technique (Sensor Medics 2400 He / CO Analyzer System, Bilthoven, Netherlands).

### Statistical analysis

Demographic and functional data were expressed as mean ± standard deviation (SD). Induced sputum cellularity was expressed as median (minimum-maximum) because of a not normal distribution. When the data showed normal distribution, they were compared with a one-way ANOVA, followed by Tukey-Kramer’s post-hoc testing. When the data did not show a normal distribution, they were compared by Kruskall-Wallis followed by Mann-Whitney testing. Correlations between variables were assessed using Spearman’s rank correlation test. A p<0.05 was considered as significant.

## Results

### Subject demographic and functional characteristics

The demographic, functional and treatment characteristics of the subjects are given in Table 2. Both disease groups were slightly older than healthy subjects. As expected both IPF and COPD had reduced FEV1 and FVC compared to healthy subjects but only COPD had a reduced FE1/FVC ratio. Total lung volume (TLC), residual lung volume (VR), lung diffusing capacity (DLCO) and transfert coefficient (KCO) were sharply reduced in the IPF group.

**Table 2:** Subject characteristics.

	Healthy subjects (n = 30)	COPD (n = 32)	IPF (n = 15)
**Age, yrs**	55(9)	63(9)***	72(9)***°
**Gender (M/F)**	11/19	24/8	12/3
**Height, cm**	169(8)	171(9)	170(12)
**Weight, Kg**	72(12)	71(17)	76(13)
**BMI, Kg/m^2^**	25(3)	24(4)	26(3)
**Smokers (Never/ex/Current), n**	13/8/9	1/17/14	2/10/3
**pack-yr**	9(13)	47(32)***	33(24)**
**FEV1%pred**	111(11)	49(16)	69(15)
**FVC %pred**	114(15)	81(14)	69(16)
**FEV1/FVC %**	80(4)	48(10)	76(11)
**TLC, %pred**	nd	115(19)	68(14)
**RV, %pred**	nd	192(58)	65(49)°°°
**DLCO %pred**	nd	56(14)	36(16)°
**KCO %pred**	nd	76(20)	59(16)
**ICS (yes/no)**	0/30	12/20	3/12
**OCS (yes/no)**	0/30	2/30	2/13

Data are expressed as mean (SD).

* p < 0.05 compared to healthy subjects.

** p < 0.001 compared to healthy subjects.

*** p < 0.0001 compared to healthy subjects.

° p < 0.05 compared to COPD.

°° p < 0.001 compared to COPD.

°°° p < 0.0001 compared to COPD.

FEV_1_: forced expiratory volume in one second; FVC: forced vital capacity; TLC: total lung capacity; RV: residual volume; DLCO: diffusion capacity of CO in the lung; ICS: inhaled corticosteroid; OCS: oral corticosteroid.

### Sputum cellularity

Induced sputum (IS) from IPF patients showed an increased cellular concentration in comparison to healthy subjects (Table 3). There was a reduction in the proportion of macrophage and lymphocyte in IPF in comparison to healthy subjects. When expressed in absolute values, neutrophils, macrophages, eosinophils and epithelial cells were all significantly increased in IPF compared to healthy subjects (Table 3). There was no significant difference between IPF and COPD.

**Table 3:** Sputum cell counts.

	*Healthy subjects*	*COPD*	*IPF*
***Sputum weight g***	*3*,*7(0*,*8–22*,*4)*	*2*,*2(0*,*4–11*,5)*	*2*,*5(0*,*8–4*,4)*
***cell x10***^***6***^ ***/g***	*0*,*8(0*,*2–7*,*6)*	*2*,*9(0*,*4–55*,9)***	*3*,*5(0*,*4–19*,5)**
***Squamous cells %***	*18(1–31)*	10(0–38)*	7(0–29)*
***Viability %***	*78(45*,*3–94*,*5)*	*70(24–100)*	*73(31–89)*
***Macrophage %***	*25(4*,*4–80*,*5)*	*9(0–77*,5)**	*16(0–41*,2)*
***x10***^***3***^ ***/g***	*203(0–6085*,*8)*	333(0–2275)**	*716(0–5891*,6)**
***Lymphocyte %***	*1*,*6(0–6*,*2)*	*1*,*5(0–6)*	*0*,*6(0–13*,2)*
***x10***^***3***^ ***/g***	*10 (0–388*,*6)*	*39(0–506)*	*14 (0–1887*,*6)*
***Neutrophil %***	*64(6*,*5–94*,*6)*	*66(11–100)*	*74(44*,*4–100)*
***x10***^***3***^ ***/g***	*423(0–5266*,*2)*	1429(129–55900)**	*2775(201*,*6–13912*,5)***
***Eosinophil %***	*0*,*2(0–5*,*8)*	2(0–81)***	*0*,*5(0–21*,*4)*
***x10***^***3***^ ***/g***	*0*,*4(0–207)*	58(0–8455)***	*17(0–4181*,6)*
***Epithelial cell %***	*2*,*9(0*,*5–25*,*3)*	*3*.*4(0–30*.*4)*	*1*,*8(0–16*,*4)*
***x10***^***3***^ ***/g***	*23(0–831*,*6)*	94(0–594)*	86(0–469)*

Results are expressed as median (min-max).

* p < 0.05 compared to healthy subjects.

** p < 0.001 compared to healthy subjects.

*** p < 0.0001 compared to healthy subjects.

### Sputum supernatant biomarkers

Several biomarkers were measured in sputum supernatant (Table 4). There was a significant increase of IGFBP-2, IL-8, TGF-β, MMP-7, MMP-9 and KL-6 (p<0.05, p<0.0001, p<0.05, p<0.05, p<0.0001, p<0.05 respectively) in IPF patients compared to HS. COPD had higher IL-6 and TNFα levels than IPF (p<0.05 and p<0.05 respectively) and HS (p<0.0001 and p<0.001 respectively) and higher IL-8 and MMP-9 than HS (p<0.0001 and p<0.001 respectively). Conversely to IL-6 and TNF-α, MMP-7 was increased in IPF compared to COPD (p<0.05).

**Table 4:** Sputum supernatant biomarkers.

	Healthy subjects	COPD	IPF
**IGF-1 (ng/ml)**	0(0–0)	0(0–0)	0(0–17)
**IGF-2 (ng/ml)**	0(0–20)	0(0–39)	0(0–24)
**IGFBP-1 (ng/ml)**	0(0–0)	0(0–0)	0(0–1)
**IGFBP-2 (ng/ml)**	30(0–120)	49(0–257)	59(3–693)*
**IGFBP-3 (ng/ml)**	167(24–1060)	196(53–1349)	222(109–786)
**IL-8 (pg/ml)**	46(0–381)	170(0–3287)***	276(46–3083)***
**TGF-β (pg/ml)**	0(0–38)	16(0–60)	32(0–88)*
**IL-6 (pg/ml)**	23(3–216)	60(20–3113)***	29(8–459)°
**IL-13 (pg/ml)**	0(0–453,2)	0(0–496,3)	0(0–1283,5)
**TNF-α (pg/ml)**	6,8(2–24,4)	14(2–489)**	10(2–88)°
**MMP-7 (ng/ml)**	20(2–93)	17(3–195)	46(0.008–190)* °
**MMP-9 (ng/ml)**	70(10–471)	264(31–969)**	415(18–970)***
**YKL-40 (ng/ml)**	8(0.8–33)	16(0.6–224)	15(0.002–204)
**KL-6 (U/ml)**	23(4–292)	44(4–975)	140(16–448)*

Results are expressed as median (min-max).

* p < 0.05 compared to healthy subjects.

** p < 0.001 compared to healthy subjects.

*** p < 0.0001 compared to healthy subjects.

° p < 0.05 compared to COPD.

°° p < 0.001 compared to COPD.

°°° p < 0.0001 compared to COPD.

### Sputum cell gene expression

**Fig 1:**
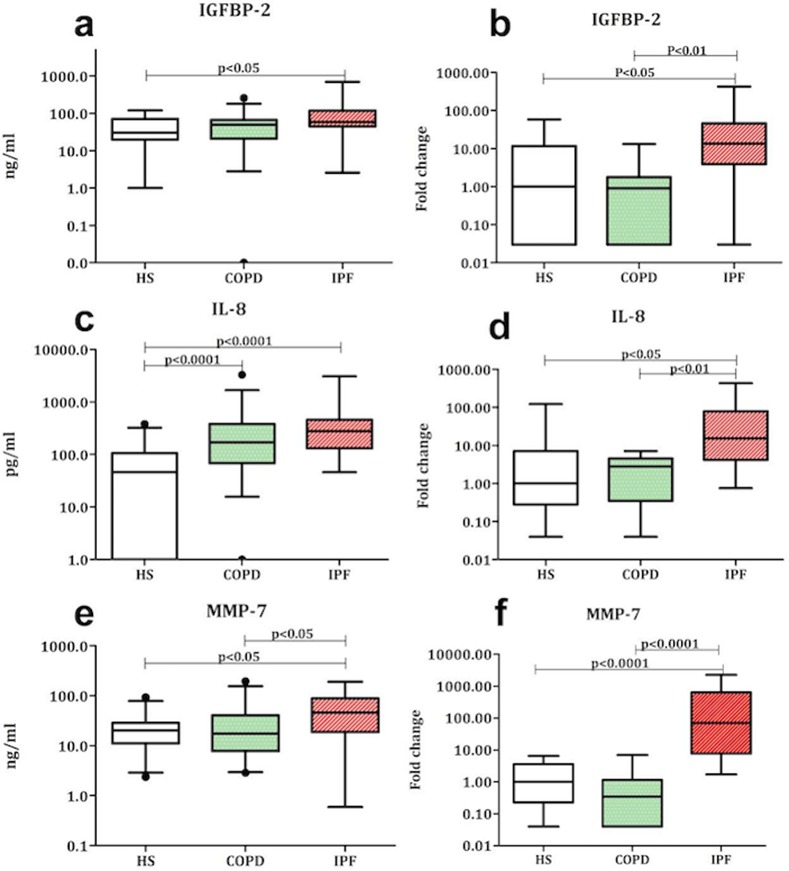
Protein and gene levels of IGFBP-2, IL-8 and MMP-7 in sputum of IPF patients. **a)** IGFBP-2 level in supernatant showing a significant increase in IPF compared to HS. **b)** Sputum cell gene expression of IGFBP-2 showing a significant increase in IPF patients compared to COPD and HS. **c)** IL-8 level in supernatant showing a significant increase in COPD and IPF compared to HS. **d)** Sputum cell gene expression of IL-8 showing a significant increase in IPF compared to COPD and HS. **e)** MMP-7 level in supernatant showing a significant increase in COPD and IPF compared to HS. **f)** Sputum cell gene expression of MMP-7 showing a significant increase in IPF compared to COPD and HS.

Gene expression analysis identified a significant increase for IGFBP-2, IL-6, IL-8 and MMP-7 in IPF compared to HS (p<0.05, p<0.01, p<0.05 and p<0.0001 respectively) and for IGFBP-2, YKL-40, IL-6, IL-8 and MMP-7 compared to COPD (p<0.01, p<0.01, p<0.05, p<0.01 and p<0.0001 respectively). (Fig 1)(Table 5). Furthermore, gene expression of TGF-β was also increased in IPF compared to COPD (p<0.001).

**Table 5:** Sputum cell gene expression.

	COPD	IPF	HS vs IPF	HS vs COPD	COPD vs IPF
	fold change	fold change	p value	p value	p value
**IGFBP-2**	0.91	13.70	0,0385	ns	0,0011
**IL-6**	4.67	24.88	0,0042	ns	0,0478
**IL-8**	2.78	15.53	0,0193	ns	0,0037
**MMP-7**	0.35	70.43	<0,0001	ns	<0,0001
**TGF-β**	0.62	5.02	ns	ns	0,0005
**TNF-α**	1.15	2.53	ns	ns	ns
**YKL-40**	0.5	5.65	ns	ns	0,0011

Data are presented as fold change relative expression to healthy subjects group. COPD: chronic obstructive pulmonary disease (n = 15); IPF: idiopathic pulmonary fibrosis (n = 12), HS: healthy subjects (n = 14).

### Biomarkers and impaired lung function

**Fig 2:**
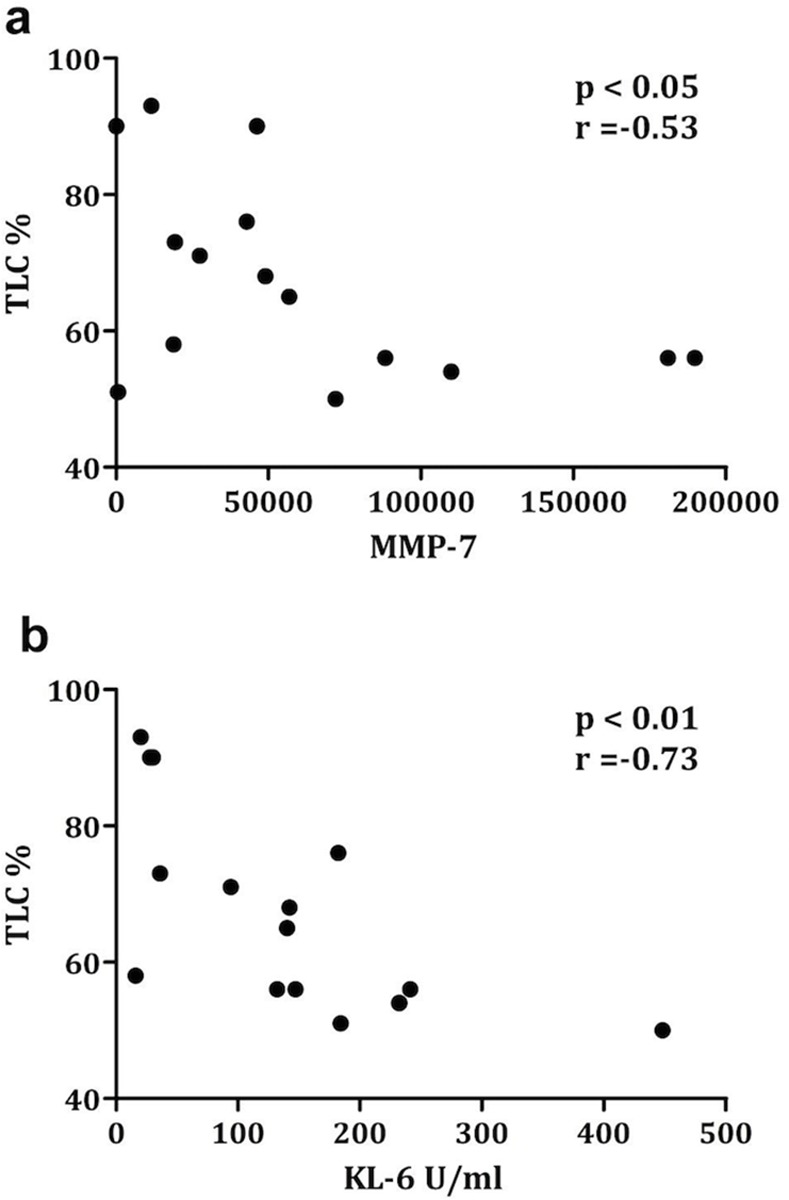
**Correlation between MMP-7 and KL-6 and TLC (% pred) in IPF. a)** Correlation between level of MMP-7 (ELISA) in supernatant of IPF patients and total lung capacity (TLC)(%predicted). **b)** Correlation between level of KL-6 (ELISA) in supernatant of IPF and TLC (%predicted).

In IPF, there was an inverse correlation between sputum supernatant protein levels of MMP-7 and KL-6 and % predicted TLC (r = -0.53, r = -0.73 respectively) (Fig 2). Diffusion lung capacity was not significantly related to any of the biomarkers.

### Relationship between age, gender and biomarkers

Our groups were not fully matched with respect to age, sex and gender and smoking status. Therefore we performed a specific analysis in the HS group in which we didn’t find any correlation between biomarkers and age, gender and smoking status (data not shown).

## Discussion

Here we report for the first time concomitant measurement of gene expression and protein levels of growth factors and matrix metalloproteinases in sputum from patients with IPF. We confirmed that TGF- β, IL-8, KL-6 and MMP-7, known to be biomarkers in IPF based on serum and BAL analysis, are also significantly higher in induced sputum from IPF patients. Furthermore IGFBP-2, recently identified as a new protein involved in IPF [14], was also significantly increased in sputum from IPF both at the gene and the protein level.

Sputum cell count of patients with IPF revealed an intense inflammatory pattern with raised total cell counts including granulocytes, macrophages and epithelial cells confirming previous finding of Beeh et al. [24]. This increased number of cells had already been described in BAL of IPF patients [25] but with a different proportion of cells, the granulocytic component being more prominent in sputum than in BAL at the expense of the lymphocyte and the macrophage fraction [2, 26]. Interestingly, in our study, IPF patients did not differ from COPD with respect to sputum cell counts. This would indicate that it is difficult to identify the main localisation of the pathological process (lung versus airways) based on the sole sputum cell count.

TGF-β, widely known as a key actor of fibrosis and known to be elevated in BAL of IPF patients [27], was increased in sputum supernatant of IPF compared to healthy subjects. Moreover gene expression of TGF-β was significantly raised in IPF compared to COPD, another disease featuring remodelling, though in the latter it mainly affects the airway compartment.

Our study also confirmed the importance of MMP-7, a matrix metalloproteinase specifically found to be raised both at gene and protein level in sputum from IPF compared to COPD and HS. While MMP-9 was increased in both IPF and COPD, it is remarkable that the raised expression and protein amount of MMP-7 was limited to patients with IPF compared to HS. Our results are in keeping with those of Zuo et al. who reported a greater MMP-7 gene expression in lung tissues of IPF patients [28] and with those of Huh et al. who reported an increased level of MMP-7 in BAL of IPF [29]. Supporting the role of MMP-7 in disease progression [17, 30], we interestingly found a negative correlation between MMP-7 levels and TLC (%pred), a physiological marker of pulmonary restriction. As our study is cross sectional it does not demonstrate that sputum MMP-7 would associate with a loss of lung volume over time but this would require a longitudinal study to confirm.

We found a significant and specific increase in KL-6 in sputum supernatant from IPF. As with MMP-7 there was also a negative correlation between TLC (%pred) and sputum protein level of KL-6. KL-6 is well known as a biomarker in pulmonary fibrosis [9, 31] and is present on the surface membrane of alveolar epithelial cells (AEC-II) and bronchiolar epithelial cells. Serum KL-6 has been previously described as predictive of acute exacerbation of IPF [9] but is not recognized as a marker of disease severity by itself. The fact that sputum levels of KL-6 relates to the loss of lung volume in our study suggests that sputum may be a more appropriate compartment than serum to evaluate the epithelial and alveolar damage occurring in IPF.

We have recently shown that serum IGFBP-2 was raised in IPF [14]. Here we extend our finding by showing that this protein was also specifically raised in sputum. The augmentation was seen both at the gene and the protein level making it very consistent. This is an original finding as the protein had not been measured in the airway/lung compartment of patients suffering from IPF before. Of interest is that gene expression of IGFBP-2 in IPF was not only increased compared to healthy subjects but also compared to COPD. There is however a previous report of increased IGFBP-2 in BAL of children with diverse interstitial lung diseases [32]. Therefore we cannot claim that IGFBP-2, though being a useful biomarker and relevant to pathophysiology [14], is specific of IPF.

Gene expression of IL-6 in IPF was increased compared to both healthy subjects and COPD though the protein level remained comparable to that measured in healthy subjects. In IPF IL-6 is pro-mitogenic for fibroblasts due to the sustained activation of MAP Kinases in contrast to what is seen normal fibroblast [33]. In order to explain that, as opposed to gene expression, protein level was not increased, we cannot rule out the possibility of a local consumption of IL-6 that participates to the fibrosing process. Conversely the high protein level of IL-6 in sputum supernatant from COPD contrasting with poor gene expression may be seen as a consequence of plasma exudation into the airways and is in keeping with our previous observation that sputum cells from COPD produced less IL-6 than those of HS [34]. Confirming previous studies [26], IL-8 was significantly increased in IPF compared to healthy subjects both at gene and at protein level while, as for IL-6, the increase in COPD was only seen at the protein level. IL-8 is known to be a strong chemotactic agent for neutrophils [35, 36] and could explain the greater number of neutrophils infiltrating the airways in IPF and COPD.

As aforementioned, one of the potential limitation of the study is the cross sectional design that precludes any interpretation of the predictive values of these biomarkers in the follow-up of the patients. A further limitation is that we were unable to assess the expression and the levels of the growth factors in patients receiving anti-fibrotic therapy. Finally the lack of perfect matching between our groups regarding age, gender and tobacco may be seen as a potential confounding factor. However we have to say that it is extremely difficult to recruit a large number of healthy subjects above the age of 65 yrs. To address this issue of potential confounding factors we have assessed the relationship between age, gender and tobacco smoking and protein and gene expression of biomarkers in the group of healthy subjects and we did not find any significant correlation.

## Conclusion

Our data show clear increase in expression and production of several growth factors and matrix metalloproteinases and chemokine in sputum from patients with IPF. Whether sputum analysis may become a suitable and less invasive tool than BAL to predict and monitor evolution of the disease and the response to treatment should be investigated in prospective longitudinal trials.

## Abbreviations

ATS: American thoracic society
BAL: Bronchoalveolar lavage
COPD: Chronic obstructive pulmonary disease
DLCO: Diffusion capacity of CO in the lung
FEV1: Forced expiratory volume in one second
FVC: Forced vital capacity
HRCT: High resolution computed tomography
IgA: Type A immunoglobulin
IGF: Insulin-like growth factor
IGFBP: Insulin-like growth factor binding protein
IL-6: Interleukine-6
IL-8: Interleukine-8
ILD: Interstitial lung disease
IPF: Idiopathic pulmonary fibrosis
KL-6: Krebs von den Lungen-6
MMP: Matrix metalloproteinase
SD: Standard deviation
SP-A: Surfactant protein-A
SP-D: Surfactant protein-D
TGF-β: Transforming growth factor beta
TLC: Total lung capacity
YKL-40: Chitinase-3-like-1, human cartilage glycoprotein-39
